# Iterative Design and Manufacturing of a Low-Cost, Low-Fidelity Simulator for Cricothyroidotomy

**DOI:** 10.7759/cureus.95585

**Published:** 2025-10-28

**Authors:** Lukas Shum-Tim, Vedanth Desaigoudar, Abdullah Mashat, Han Hsiao, Peter R.A. Malik, Kevin R Malone, Mohamed H Abdalla, Shrivatsan Rajagopalan, Nicolas Mourad, Cecily Bos, Emilie Joos, Shahrzad Joharifard

**Affiliations:** 1 Faculty of Health Sciences, Department of Surgery, Division of General Surgery, McMaster University, Hamilton, CAN; 2 Department of General Surgery, Niagara Health Knowledge Institute, Niagara, CAN; 3 Branch for Global Surgical Care, Department of Surgery, Global Surgery Lab, Vancouver, CAN; 4 Faculty of Engineering, University of British Columbia, Vancouver, CAN; 5 Department of Surgery, University of British Columbia, Vancouver, CAN; 6 Faculty of Medicine, University of British Columbia, Vancouver, CAN; 7 Faculty of Medicine and Health Sciences, McGill University, Montreal, CAN; 8 Lawrence S. Bloomberg Faculty of Nursing, University of Toronto, Toronto, CAN; 9 Faculty of Medicine, Ains Shams University, Cairo, EGY; 10 Department of Surgery, Division of Trauma and Acute Care Surgery, University of British Columbia, Vancouver, CAN; 11 Department of Surgery, Division of Pediatric Surgery, University of British Columbia, British Columbia Children’s Hospital, Vancouver, CAN

**Keywords:** emergency surgery, general surgery, global surgery, head and neck surgery, surgical airway, surgical education, surgical simulation, trauma surgery

## Abstract

In a trauma setting, cricothyroidotomy is a life-saving procedure performed to secure a compromised airway. The rarity and high-stakes nature of the procedure drastically limit training opportunities. Existing models are expensive and inaccessible. Our objective was to design a low-cost, low-fidelity cricothyroidotomy simulator using universally available materials to ensure widespread access. Our technical report used an iterative design-based research methodology. A multidisciplinary team first determined the essential steps of a cricothyroidotomy. We then established the anatomical components required to perform the procedure: skin, subcutaneous tissue, trachea, thyroid cartilage, and cricothyroid membrane. Multiple prototypes built from a variety of materials were tested by performing simulated cricothyroidotomies. Our cricothyroidotomy simulator requires less than five minutes to construct and costs $0.53 USD. The trachea is made from a plastic water bottle. An egg carton mimics the thyroid cartilage. A nitrile glove slides onto the trachea to simulate the cricothyroid membrane. Slime, created using a mixture of glue, detergent, sodium bicarbonate, and cornstarch, is used for the skin and subcutaneous tissues. Each component can be used infinitely, except for the nitrile glove, which needs to be replaced after 10 uses. Our cricothyroidotomy simulator can be used to train healthcare providers in both well-resourced and resource-limited settings. In addition to making our design blueprints open access, we have intentionally chosen widely accessible and eco-friendly materials to make our simulator available worldwide.

## Introduction

Traumatic injuries requiring treatment represented 19.1% of the global burden of disease in 2021, with 4.3 million cases leading to death [1]. Low and middle-income countries (LMICs) are disproportionately affected [2]. Although initiatives such as the World Health Organization (WHO) Global Emergency and Trauma Care Initiative (GETI) strive to increase capacity for quality emergency care [3], the lack of access to surgical care, especially in LMICs, is a significant contributing factor to global morbidity and mortality [4].

During a trauma resuscitation, airway compromise can severely restrict airflow and negatively impact a patient’s outcome. If the airway cannot be secured non-invasively, a surgical airway must be created through a procedure called cricothyroidotomy. When untrained, non-surgeon physicians perform this procedure, complication rates can be as high as 31% [5]. With training, non-surgeon physicians can perform a cricothyroidotomy with complication rates similar to those of specialized surgeons [5].

Unfortunately, existing cricothyroidotomy simulators are expensive to purchase, with costs ranging from hundreds to thousands of dollars per simulator or per replacement kit [6,7]. These high acquisition and per-use costs make these training tools inaccessible in low-resource settings.

This technical report provides a comprehensive overview of our cricothyroid training simulator’s design and manufacturing process, covering its specifications, step-by-step assembly process, and cost analysis.

This article was previously presented as a meeting poster presentation at the 2024 CCGH Canadian Conference for Global Health on October 25, 2024.

## Technical report

Conceptual design

This technical report used an iterative design-based research methodology with a multidisciplinary, collaborative approach between medical students, surgical residents, surgeons, nurses, and biomedical engineers from five different institutions. The team divided the upper airway anatomy into the essential elements that the simulator would need to replicate: skin, subcutaneous tissue, trachea, thyroid cartilage, and cricothyroid membrane. The simulator needed to include anatomical landmarks, permit incision of the cricothyroid membrane, allow digital confirmation of airway entry, and enable placement of a tracheostomy or endotracheal tube into the simulated trachea.

The final design must allow trainees to ‘‘show how’’ a cricothyroidotomy is performed rather than simply enumerating the steps, taking Miller’s pyramid into account as a conceptual framework [8]. This design element enables the demonstration of how to perform a cricothyroidotomy on a simulator in a non-pressured learning environment, which can increase the number of supervised repetitions and provide a space for error, a crucial aspect for learning this high-acuity, low-opportunity procedure [9,10].

**Figure 1:**
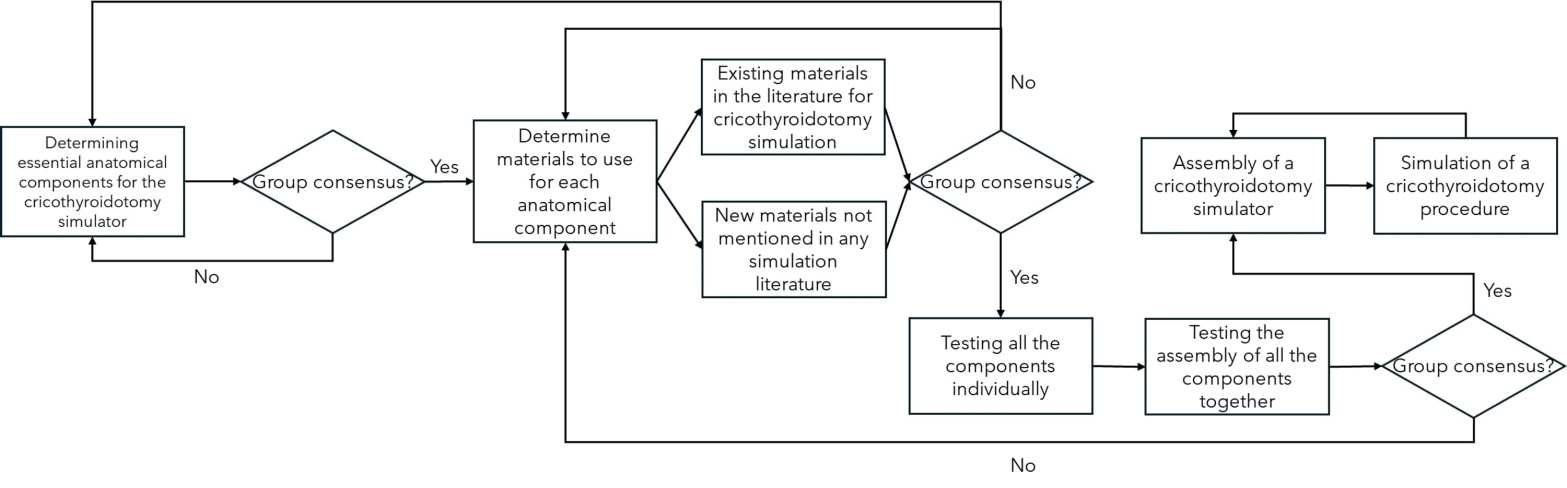
Cricothyroidotomy simulator development process in a schematic form.

Existing cricothyroidotomy models were used as a starting point for developing our simulator [11-13]. Materials for each anatomical component were identified, procured, and evaluated for suitability based on accessibility, cost, and functional realism. The development process involved five iterative prototype designs over several months, as depicted in Figure 1.

Material selection and prototype iterations

The first prototype incorporated three-dimensional (3D)-printed airway structures to replicate the relevant anatomy. However, this approach was excluded due to the limited accessibility of 3D printers, particularly in low-resource settings. The second prototype utilized organic materials, such as pasta dough, to simulate skin. While the material cost was negligible and the components were biodegradable, the model’s construction time was excessive, rendering it impractical for repeated educational use.

The third prototype employed plastic tubing to represent the trachea, a square-cut cardboard segment for the thyroid cartilage, and adhesive tape for the cricothyroid membrane. Although this iteration marked progress in structural representation, it lacked both a skin layer and a stable base. The fourth prototype addressed these limitations by adding Play-Doh as a skin analog and incorporating an egg carton as a base to stabilize the model. While the Play-Doh was reusable and easy to mold, it failed to replicate the tactile feeling encountered during skin incision adequately. Further, Play-Doh is not globally accessible and was thus not the ideal material for our project.

Assembly procedure

**Figure 2:**
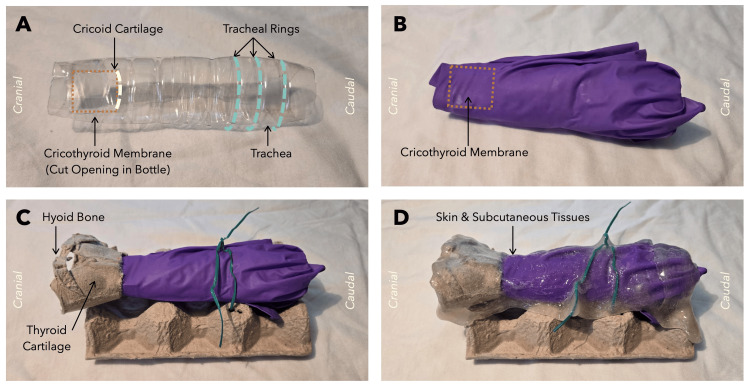
Assembly of the cricothyroidotomy simulator. Panel A: A water bottle cut and folded on itself, mimicking the trachea. An opening was cut anteriorly to create the cricothyroid cartilage and space for the cricothyroid membrane to rest on (orange dotted square). The inferior edge of the cut opening represents the cricoid cartilage (pale dotted line). The water bottle grooves represent the tracheal rings (green dotted lines). Panel B: A purple nitrile glove was added over the water bottle trachea, mimicking the cricothyroid membrane (orange dotted square). Panel C: A single egg carton container was added anterior to the water bottle trachea, mimicking the hyoid bone and thyroid cartilage. The simulator is secured on multiple egg carton containers with a green metal wire to stabilize it. Panel D: Slime was added anterior to the simulator, mimicking skin and subcutaneous tissue.

The assembly of the final prototype is illustrated in Figure 2. Panel A depicts a 500 mL plastic water bottle that has been cut at the cranial aspect and secured with transparent adhesive tape to simulate the tubular structure of the trachea. The bottle’s grooves emulate the tracheal ring, while the cut opening represents the cricoid membrane. The caudal aspect of the cut opening represents the cricoid cartilage. Panel B shows a nitrile glove stretched over the bottle opening to simulate the cricothyroid membrane. Panel C illustrates the placement of an egg carton at the cranial aspect of the bottle, shaped to mimic the thyroid cartilage and hyoid bone. The remaining portion of the egg carton serves as a structural base, stabilized with a metal wire “twist tie” to maintain the model’s integrity. Panel D demonstrates the addition of a synthetic soft-tissue analog (“slime”), applied anteriorly to simulate the skin and subcutaneous tissue. This material is comprised of liquid glue, baking soda, laundry detergent, and cornstarch.

Results

The simulator can be assembled in under five minutes. The completed model weighs approximately 70 g and has dimensions of 5 × 5 × 16 cm. A summary table outlining the materials tested, their corresponding anatomical analogs, and the associated advantages and disadvantages is provided in Appendix 1. Detailed assembly instructions are presented in Appendix 2. We have also provided additional assembly instructions in a video format, which is available in Appendix 3. A full cost breakdown is presented in Appendix 4.

The cost of constructing a single cricothyroidotomy simulator was $0.53 USD. The cost-per-use was approximately $0.05 USD, primarily due to replacing the nitrile glove component, which is reusable up to 10 times. This estimate excludes the cost of procedural instruments required to perform the cricothyroidotomy itself (e.g., scalpel, endotracheal tube, or tracheostomy tube).

## Discussion

We developed a low-cost, low-fidelity cricothyroidotomy simulator constructed from widely accessible materials, which can be adapted based on local availability (see Appendix 1 for alternative materials). The model is anatomically customizable, allowing for replicating variations such as adult versus pediatric size or male versus female larynx. It is lightweight, portable, and rapidly assembled without requiring specialized skills or equipment.

Notably, all simulator components are recyclable or biodegradable, supporting their use as a sustainable training tool. For instance, the water bottle and the metal wire can be recycled, while the egg carton, the slime, and the latex glove are biodegradable. This reduces the environmental impact compared to the current commercially available models, which use single-use plastics for the cricothyroid membranes [6,7].

Given the substantial cost of commercially available cricothyroidotomy simulators, this model offers a cost-effective alternative. Existing low-cost simulators in the literature are often associated with higher per-unit or per-use costs [13] or rely on technologies such as 3D printing, which may limit global applicability [14,15]. While cadaveric models could provide higher fidelity, their use is constrained by low availability, high cost, and restricted time frame [16]. An alternative would be using animal cadaveric models instead, but cricothyroidotomy training on such models has not been shown to be better than on a human model [17].

A comparable simulator utilizing similarly accessible materials with equivalent assembly time has been previously described [11]. However, a distinguishing feature of our model is the incorporation of “slime” as a reusable subcutaneous tissue analog. Slime, a non-Newtonian fluid commonly used in educational settings, is primarily composed of polyvinyl acetate, a safe, biodegradable compound [18], and activated with boric acid-containing agents, such as laundry detergent or contact lens solution [19]. To our knowledge, the use of slime in cricothyroidotomy simulation has not been previously reported. The material can be reshaped and reused following incision, similar to bread dough or commercially purchased Play-Doh. It must be stored in an airtight container to prevent desiccation.

Limitations

Low-fidelity cricothyroidotomy simulation has demonstrated effectiveness in training healthcare providers, with outcomes comparable to those trained using high-fidelity models [11,20]. While novice learners may derive greater benefit from low-fidelity simulations and more experienced trainees may respond better to higher fidelity, the observed differences in knowledge acquisition and procedural skill development are not statistically significant [21]. Despite this, participants frequently prefer high-fidelity simulators, although they demand substantially greater financial and logistical resources [22].

Low-fidelity models, by contrast, are more cost-effective and accessible, making them a practical alternative in many educational settings [11,20-22]. The selection of simulation fidelity should be guided by specific learning objectives, available resources, and the broader training context to optimize educational outcomes. In this regard, our low-fidelity simulator is particularly well-suited for training healthcare providers in resource-limited environments. Although the model was designed and manufactured in a high-resource environment, the design process was informed by team members’ lived and professional experiences with lower resource settings.

## Conclusions

We developed a low-cost cricothyroidotomy simulator that uses inexpensive materials available worldwide. Our goal is to remove barriers to cricothyroidotomy training so that patients presenting with airway compromise can access surgical care, regardless of where they are treated. Our next step is to validate the face, content, and construct validity of our simulator.

## Appendices

Appendix 1: Proposed materials to simulate anatomical structures involved in a cricothyroidotomy

**Table 1:** Proposed materials to simulate anatomical structures involved in a cricothyroidotomy. ABD: abdominal; PVC: polyvinyl chloride; 3D: three-dimensional; LMIC: low and middle-income countries

Material	Anatomical structure	Advantages	Disadvantages
Pasta (cooked lasagna)	Skin/Subcutaneous tissue	Mimics texture well	Difficult to reuse
		Easy to acquire	Difficult to preserve
		Can be made from basic materials (water, flour)	Can break relatively easily
		Low cost	Short shelf-life once cooked
		Biodegradable	Requires access to a heat source for an extended period (time-intensive)
Silicone gel	Skin/Subcutaneous tissue	Standard material to mimic tissue in other simulators	Can be challenging to acquire in LMICs
		Can be molded into an appropriate shape using a 3D-printed mold	Costly
			Takes time to prepare the cast
Play-Doh	Skin/Subcutaneous tissue	Can be molded into an appropriate shape	Not always readily accessible in LMICs
		Flexible, can mimic texture appropriately	
		Low cost	Cutting through with a scalpel feels like cutting through (bread) dough
		Can be reused infinitely	
Felt fabric	Skin/Subcutaneous tissue	Can be shaped into what is needed	Difficult to cut
			Not readily accessible
			Does not mimic skin well
Slime	Skin/Subcutaneous tissue	Can be molded into an appropriate shape	Can be messy to make
		Flexible, can mimic texture appropriately	
		Low cost	Needs to be wrapped in a plastic bag after use to prevent drying
		Can be reused infinitely	
		Multiple substitute ingredients possible, can use local ingredients	
ABD pad/gauze	Skin/Subcutaneous tissue	Should be accessible according to the World Health Organization	Difficult to cut
			Difficult to stabilize
Adhesive tape	Cricothyroid membrane	Easy to acquire	Can tear in unintended ways
		Easy to place over the trachea	Not reusable
		Easy to swap	
Parchment paper	Cricothyroid membrane	Low cost	Too brittle for a membrane
			Hard to maintain in place
Elastic	Cricothyroid membrane	Low cost	Difficult to cut
		Readily accessible	Difficult to insert an endotracheal tube through it
		Easy to place around the trachea	Can deform the trachea
Plastic pellicle	Cricothyroid membrane	Low cost	Difficult to stabilize on the trachea
		Easy to cut	
		Can be substituted for a plastic bag	
Nitrile glove	Cricothyroid membrane	Low cost	Difficult to fold and keep it in place
		Can be reused a few times	
		Does not need glue to stabilize on the trachea	
		Should be accessible according to the World Health Organization	
Paper maché	Thyroid cartilage	Easy to make (glue, newspaper, water)	Takes time to make
		Easy to carve into the appropriate shape	
		Solid texture	Can be messy
		Low cost	
Egg carton	Thyroid cartilage	Easy to carve	Accessibility could be an issue
		Solid enough to mimic cartilage	
		Biodegradable	
		Low cost	
		Can be obtained from recycled material	
Menstrual cup	Thyroid cartilage	Can be carved into an appropriate shape	Can be difficult to access
		Firm, can mimic thyroid cartilage well	Expensive
3D printed	Thyroid cartilage, trachea	Easy to find templates online	Expensive
		Templates may be modified to fit particular anatomy	Not everyone has a 3D printer
Leek	Trachea	Can be grown almost anywhere (needs water, light, and seeds)	Difficult to reuse
		Cheap	Difficult to preserve
		Biodegradable	Short shelf life
			Takes time to grow
Hardened bread	Trachea	Easy to acquire	Difficult to reuse
		Can be made from basic materials (water, flour)	Difficult to preserve
		Cheap	Can break relatively easily
		Biodegradable	Short shelf life
Sponge	Trachea	Can be carved into an appropriate shape	Too soft to simulate adult cartilage
PVC pipe	Trachea	Premade tube-like structure	Too hard to simulate adult cartilage
		Can carve tracheal rings	Not readily accessible
			Can become expensive
Rubber hose	Trachea	Can come from a perforated hose (recycled material)	Can be expensive if not from a recycled source
		Hard, but flexible enough, can mimic cartilage	
Ventilator tube	Trachea	Has grooves for tracheal rings	Expensive
		Flexible, mimics cartilage well	Not accessible everywhere
Water bottle	Trachea	Can have grooves to mimic tracheal rings	Needs glue to keep an assigned shape (has a lot of memory)
		Mimics cartilage well	
		Can be shaped into a trachea-like tube	
		Low cost	
		Accessible worldwide	
		Recyclable	

Appendix 2: Assembly instructions

**Table 2:** Assembly instructions for the cricothyroidotomy simulator according to each general assembly step.

General assembly steps	Assembly instructions for each general assembly step
Part 1: Preparing the water bottle (trachea)	1. Use a sharp object (such as a scalpel or scissors) to cut out the bottle’s top and bottom portions (curved parts). The main piece should be a cylinder with both sides open
	2. Make two vertical incisions (the height) along the radial axis of the bottle to get two semi-circular pieces (should look like two long floating boats)
	3. If any moisture is present, use a cloth to dry it
	4. Fold one piece on itself to create a cylindrical tube (with a small radius and two open ends). The second semi-circular piece can be used for a second model
	5. Using the scotch tape, secure the cylindrical shape of the tube in place
	6. Cut out a small square (1.2 cm in length and width) approximately 1.5 cm below one opening of the cylindrical tube
Part 2: Preparing egg carton (thyroid cartilage + bone)	1. Using a sharp object (such as a scalpel), cut out one egg compartment
	2. Cut a curvilinear part of the egg compartment
	3. Using glue, attach this part around the ring of the larger surface of the egg compartment to form the thyroid bone on top of the thyroid notch
	4. Using a sharp object, make a circular cut to remove the inferior surface of the egg carton compartment. Both the top and bottom should be open
Part 3: Preparing the nitrile glove (cricothyroid membrane)	1. Take the cylindrical water bottle tube created in Part 1, and place it in any finger pockets of the nitrile glove
	2. Wrap the remaining finger pockets and base of the glove around the circumference of the cylindrical tube as tightly as possible. Consider placing the remaining parts of the glove inside the bottom tube surface
Part 4: Preparing slime for the skin/subcutaneous tissue	1. Activate the baking soda by mixing ¼ teaspoon of baking soda with one teaspoon of water and keep it aside
	2. Mix eight teaspoons of liquid glue thoroughly with two teaspoons of laundry detergent
	3. While mixing, add the activated baking soda mixture slowly to form the wet base of the slime
	4. Combine this wet mixture with one tablespoon of cornstarch and knead thoroughly using your hands to form the stretchy, reusable slime
Part 5: Assembling the trachea model using water bottle + egg compartment	1. Take the combined nitrile glove + water bottle cylindrical pieces and place them through the bottom surface of the egg compartment
	2. Next, place this on top of a flat piece of carton (can be the cover of the egg carton)
	3. Place the slime on top of the model
	4. Secure it in place using a metal wire

Appendix 3: Additional assembly instructions

Appendix 4: Material cost breakdown

**Table 3:** Cost breakdown of each material used in USD and the total cost per model. USD: United States Dollars ($)

Material used	Cost ($)
Plastic water bottle	0.10
Egg carton	0.07
Nitrile glove	0.09
Washable white glue	0.13
Corn starch	0.09
Baking soda	0.005
Laundry detergent	0.03
Scotch tape	0.0009
Metal wire	0.000275
Plastic water bottle	0.10
Total cost per model	0.53
